# Trends in HIV/AIDS morbidity and mortality in Eastern Mediterranean countries, 1990–2015: findings from the Global Burden of Disease 2015 study

**DOI:** 10.1007/s00038-017-1023-0

**Published:** 2017-08-03

**Authors:** GBD 2015 Eastern Mediterranean Region HIV/AIDS Collaborators, Charbel El Bcheraoui, Haidong Wang, Raghid Charara, Ibrahim Khalil, Maziar Moradi-Lakeh, Ashkan Afshin, Michael Collison, Farah Daoud, Adrienne Chew, Kristopher J. Krohn, Austin Carter, Kyle J. Foreman, Fei He, Nicholas J. Kassebaum, Michael Kutz, Mojde Mirarefin, Grant Nguyen, Naris Silpakit, Amber Sligar, Amanuel A. Abajobir, Kalkidan H. Abate, Kaja M. Abbas, Foad Abd-Allah, Semaw F. Abera, Kelemework Adane, Arnav Agarwal, Aliasghar Ahmad Kiadaliri, Alireza Ahmadi, Muktar B. Ahmed, Faris H. Al Lami, Khurshid Alam, Deena Alasfoor, Reza Alizadeh-Navaei, Fatma Al-Maskari, Rajaa Al-Raddadi, Khalid A. Altirkawi, Nelson Alvis-Guzman, Walid Ammar, Nahla Anber, Carl Abelardo T. Antonio, Palwasha Anwari, Hamid Asayesh, Rana J. Asghar, Tesfay Mehari Atey, Euripide F. G. A. Avokpaho, Tadesse Awoke Ayele, Peter Azzopardi, Umar Bacha, Aleksandra Barac, Till Bärnighausen, Shahrzad Bazargan-Hejazi, Neeraj Bedi, Isabela M. Bensenor, Adugnaw Berhane, Pascal O. Bessong, Addisu S. Beyene, Zulfiqar A. Bhutta, Charles Birungi, Zahid A. Butt, Lucero Cahuana-Hurtado, Hadi Danawi, José das Neves, Kebede Deribe, Amare Deribew, Don C. Des Jarlais, Samath D. Dharmaratne, Shirin Djalalinia, Kerrie E. Doyle, Aman Y. Endries, Babak Eshrati, Emerito Jose A. Faraon, Maryam S. Farvid, Seyed- Mohammad Fereshtehnejad, Tesfaye R. Feyissa, Florian Fischer, Alberto L. Garcia-Basteiro, Tsegaye T. Gebrehiwot, Hailay A. Gesesew, Melkamu Dedefo Gishu, Elizabeth Glaser, Philimon N. Gona, Harish C. Gugnani, Rahul Gupta, Hassan Haghparast Bidgoli, Gessessew B. Hailu, Randah R. Hamadeh, Mitiku T. Hambisa, Samer Hamidi, Hilda L. Harb, Habtamu A. Hareri, Nobuyuki Horita, Abdullatif Husseini, Ahmed Ibrahim, Spencer L. James, Jost B. Jonas, Amir Kasaeian, Nigussie A. Kassaw, Yousef S. Khader, Ejaz A. Khan, Gulfaraz Khan, Abdullah T. A. Khoja, Jagdish Khubchandani, Yun Jin Kim, Ai Koyanagi, Barthelemy Kuate Defo, Heidi J. Larson, Asma A. Latif, Cheru T. Leshargie, Raimundas Lunevicius, Mohammed Magdy Abd El Razek, Reza Majdzadeh, Azeem Majeed, Reza Malekzadeh, Tsegahun Manyazewal, Desalegn Markos, Habibolah Masoudi Farid, Alem Mehari, Alemayehu B. Mekonnen, Peter Memiah, Ziad A. Memish, Walter Mendoza, Melkamu M. Mengesha, Desalegn T. Mengistu, Haftay B. Mezgebe, Francis A. Mhimbira, Ted R. Miller, Ami R. Moore, Ghina R. Mumtaz, Gopalakrishnan Natarajan, Joel Negin, Carla M. Obermeyer, Felix A. Ogbo, In-Hwan Oh, Erika Ota, David M. Pereira, Farshad Pourmalek, Mostafa Qorbani, Amir Radfar, Anwar Rafay, Vafa Rahimi-Movaghar, Rajesh Kumar Rai, Usha Ram, David L. Rawaf, Salman Rawaf, Andre M. N. Renzaho, Satar Rezaei, Mohammad Sadegh Rezai, Hirbo S. Roba, Gholamreza Roshandel, Mahdi Safdarian, Saeid Safiri, Mohammad Ali Sahraian, Payman Salamati, Abdallah M. Samy, Benn Sartorius, Sadaf G. Sepanlou, Masood A. Shaikh, Morteza Shamsizadeh, Ephrem L. Sibamo, Jasvinder A. Singh, Badr H. A. Sobaih, Sergey Soshnikov, Muawiyyah B. Sufiyan, Bryan L. Sykes, Nuno Taveira, Teketo K. Tegegne, Arash Tehrani-Banihashemi, Tesfalidet Tekelab, Girma Temam Shifa, Mohamad-Hani Temsah, Belay Tesssema, Roman Topor-Madry, Kingsley N. Ukwaja, Olalekan A. Uthman, Stein Emil Vollset, Fiseha Wadilo, Tolassa Wakayo, Minyahil A. Woldu, Abdulhalik Workicho, Shimelash B. Workie, Mohsen Yaghoubi, Ayalnesh Z. Yalew, Hassen H. Yimam, Naohiro Yonemoto, Seok-Jun Yoon, Marcel Yotebieng, Mustafa Z. Younis, Maysaa E. S. Zaki, Aisha O. Jumaan, Theo Vos, Simon I. Hay, Mohsen Naghavi, Christopher J. L. Murray, Ali H. Mokdad

**Affiliations:** Institute for Health Metrics and Evaluation, 2301 5th Avenue, Suite 600, Seattle, WA 98121 USA

**Keywords:** HIV, HIV mortality, Eastern Mediterranean Region, Burden of disease

## Abstract

**Objectives:**

We used the results of the Global Burden of Disease 2015 study to estimate trends of HIV/AIDS burden in Eastern Mediterranean Region (EMR) countries between 1990 and 2015.

**Methods:**

Tailored estimation methods were used to produce final estimates of mortality. Years of life lost (YLLs) were calculated by multiplying the mortality rate by population by age-specific life expectancy. Years lived with disability (YLDs) were computed as the prevalence of a sequela multiplied by its disability weight.

**Results:**

In 2015, the rate of HIV/AIDS deaths in the EMR was 1.8 (1.4–2.5) per 100,000 population, a 43% increase from 1990 (0.3; 0.2–0.8). Consequently, the rate of YLLs due to HIV/AIDS increased from 15.3 (7.6–36.2) per 100,000 in 1990 to 81.9 (65.3–114.4) in 2015. The rate of YLDs increased from 1.3 (0.6–3.1) in 1990 to 4.4 (2.7–6.6) in 2015.

**Conclusions:**

HIV/AIDS morbidity and mortality increased in the EMR since 1990. To reverse this trend and achieve epidemic control, EMR countries should strengthen HIV surveillance, and scale up HIV antiretroviral therapy and comprehensive prevention services.

**Electronic supplementary material:**

The online version of this article (doi:10.1007/s00038-017-1023-0) contains supplementary material, which is available to authorized users.

## Introduction

In 2015, HIV/AIDS was the 12th-leading cause of death worldwide after being the eighth in 2005 when the epidemic peaked (Institute for Health Metrics and Evaluation (IHME) 2017). More than 1.2 million people are estimated to have died in 2015 due to HIV/AIDS despite the considerable achievements in HIV care since the late 1980s (Wang et al. 2016b). This reflects the challenges faced by public health policymakers and program managers, health professionals, and the global community in dealing with this epidemic.

The burden of the HIV/AIDS epidemic has rapidly changed since the 1990s with the introduction of HIV antiretroviral therapy (ART) and other effective interventions (UNAIDS 2015). While incidence has declined continuously since the mid-1990s, mortality continued to rise and peaked in 2005 at 1.8 million deaths worldwide (Wang et al. 2016b). Inspired by the successes of responding to AIDS, global leaders have committed to and embarked on ending the AIDS epidemic as a public health threat by 2030, without leaving anyone behind (UNAIDS 2014a). Today, there are large variations in incidence and mortality between regions and countries (Wang et al. 2016b). In the Eastern Mediterranean Region (EMR), and despite recent progress (Institute for Health Metrics and Evaluation (IHME) 2017), estimates of HIV/AIDS continue to be challenged with limitations in data availability and by insufficient epidemiological surveillance among those most at-risk of infection (Shawky et al. 2009; Mumtaz et al. 2014a). The EMR has a population of about 583 million people. Countries in the EMR vary significantly in terms of their gross domestic product, socio-demographic profiles, health indicators, and health system capacities and coverage (WHO EMRO 2017).

The EMR has several vulnerability factors for HIV (Abu-Raddad et al. 2010). The socio-cultural and socioeconomic fabric as well as the demographic structure of the region is evolving rapidly (Abu-Raddad et al. 2010). Extensive levels of migration, displacement, mobility, and conflicts are a hallmark of the region (UNAIDS RST MENA 2008). Injection drug use is also a major challenge in a region that produces most of the world’s supply of heroin and is at the crossroads of major drug trade routes (UNODC 2007).

The emerging HIV epidemics among the most at-risk populations, such as men who have sex with men (MSM) and people who inject drugs (PWID), constitute the main feature of HIV epidemiology in the EMR today within a context that criminalizes and marginalizes these populations (Simmons 2014; Mumtaz et al. 2014a). The majority of these epidemics are recent, having emerged within the last two decades (Mumtaz et al. 2014b). In addition to these documented epidemics, there is evidence suggesting hidden, undetected epidemics among the most at-risk populations in countries with still weak HIV surveillance systems (Mumtaz et al. 2014a).

Data on disability and mortality from HIV are crucial in understanding the regional response to the disease. To inform HIV policy, programming, and resource allocation about the state of the epidemic in EMR countries, we used the results of the GBD 2015 study to report the HIV/AIDS burden in these countries.

## Methods

The Eastern Mediterranean Region (EMR) countries, based on the World Health Organization classification, are the Islamic Republic of Afghanistan, the Kingdom of Bahrain, Djibouti, the Arab Republic of Egypt, the Islamic Republic of Iran, the Republic of Iraq, the Hashemite Kingdom of Jordan, the State of Kuwait, Lebanon, the State of Libya, the Kingdom of Morocco, the Sultanate of Oman, the Islamic Republic of Pakistan, Palestine, the State of Qatar, the Kingdom of Saudi Arabia, the Federal Republic of Somalia, the Republic of Sudan, the Syrian Arab Republic, the Republic of Tunisia, the United Arab Emirates, and the Republic of Yemen.

A detailed methodology of HIV/AIDS mortality estimation for GBD 2015 has been published elsewhere (Wang et al. 2016b). We used all available data sources including vital registration, verbal autopsies, surveys, publications, and reports. These data sources have been published elsewhere as an appendix (Wang et al. 2016b), and are available from the Global Health Data Exchange (Institute for Health Metrics and Evaluation 2017). Briefly, the GBD estimation framework contains three sources for estimates of HIV-specific mortality: estimated HIV mortality from Spectrum (Brown et al. 2014; Stover et al. 2014); estimated excess HIV/AIDS mortality in our all-cause mortality estimation process; and spatiotemporal Gaussian process regression smoothed cause-specific HIV/AIDS mortality from vital registration (VR) systems that were adjusted for incompleteness and misclassification of causes of death (Wang et al. 2016a). Tailored estimation methods were used to produce final estimates of mortality depending on age groups and the availability and quality of data for mortality of HIV/AIDS.

Years of life lost (YLLs) were calculated by multiplying the mortality rate by population by age-specific life expectancy from the reference life table used in the GBD study. Years lived with disability (YLDs) were computed as the prevalence of a sequela multiplied by the disability weight for that sequela without age weighting or discounting. The YLDs arising from HIV/AIDS are the sum of the YLDs for each of the sequelae associated with HIV/AIDS. Disability-adjusted life years (DALYs) are computed as the sum of YLLs and YLDs. Detailed methods on YLLs, YLDs, and DALYs are published elsewhere (GBD 2015 DALYs and HALE Collaborators 2016; GBD 2015 Disease and Injury Incidence and Prevalence Collaborators 2016; GBD 2015 Risk Factors Collaborators 2016).

We estimated incidence and prevalence from the recoded spectrum model. This model was updated with assumptions of on-ART and off-ART mortality, as well as other program data available from the UNAIDS country files. Vital registration systems and sample registration systems provide some of the most reliable sources for estimation of HIV cause-specific deaths. Later, our cohort incidence bias adjustment method was used to scale the sizes of each incidence cohort on the basis of the raw estimates of HIV mortality from spectrum, adjusted for incompleteness and cause misclassification using unadjusted incidence curves and those observed in the vital registration system (Wang et al. 2016a). More details about this method have been published previously (Wang et al. 2016b).

We also estimated risk factors following the GBD study’s comparative assessment of risk factors detailed elsewhere (Forouzanfar et al. 2015). Briefly, this method uses data for excess mortality and disability associated with risk factors, data for exposure to risks, and evidence-based assumptions on the desired counterfactual distribution of risk exposure. The attributable burden of a risk factor is estimated by multiplying DALYs from HIV/AIDS by the population attributable fraction for HIV/AIDS due to that risk factor.

We report age-standardized estimates, and 95% uncertainty intervals (UI) for each estimate—such as rates or numbers of deaths or DALYs. We estimated UIs by taking 1000 samples from the posterior distribution of each quantity and using the 25th- and 975th-ordered draws of the uncertainty distribution (Wang et al. 2016a). For 2015, we estimated the expected burden for each of the three measures (mortality, YLLs, and YLDs) as a function of each country’s Socio-demographic Index (SDI)—a composite measure based on levels of income—education, and fertility (Wang et al. 2016a). SDI was developed for GBD 2015 to provide an interpretable synthesis of overall development, as measured by lag-dependent income per capita, average educational attainment in the population over 15 years of age, and total fertility rates. In GBD 2015, SDI was computed by rescaling each component to a scale of zero to one, with zero being the lowest observed educational attainment, lowest income per capita, and highest fertility rate from 1980 to 2015, and one being the highest observed educational attainment, highest income per capita, and lowest fertility rate during that time, and then taking the geometric mean of these values for each location-year.

### Role of the funding source

The funder of the study had no role in study design, data collection, data analysis, data interpretation, or writing of the report.

## Results

### Mortality

**Fig. 1:**
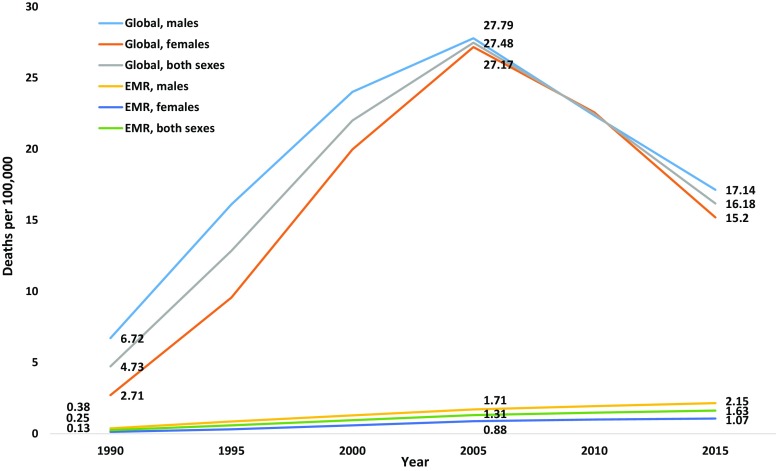
Trends of HIV/AIDS age-standardized mortality worldwide, and in the Eastern Mediterranean Region (EMR), 1990–2015. (Global Burden of Disease Study 2015, Global, the Eastern Mediterranean Region, 1990–2015)

The proportion of deaths attributable to HIV/AIDS has increased steadily in the EMR since 1990 by 6.7% annually (Fig. 1).

**Fig. 2:**
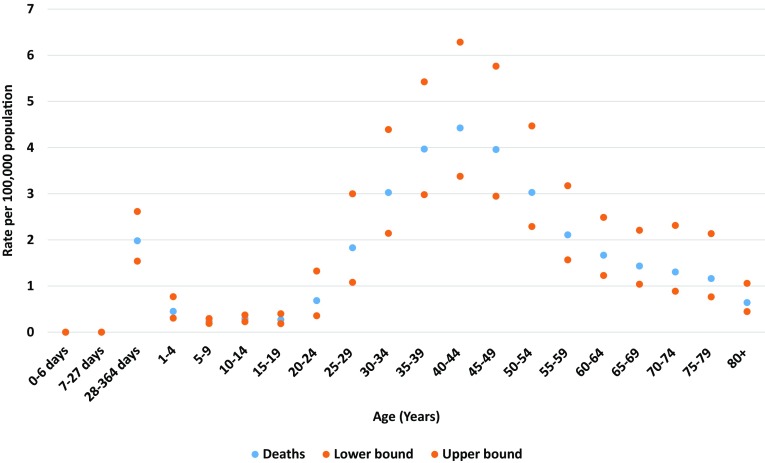
Distribution of HIV/AIDS mortality rate and 95% uncertainty levels in the Eastern Mediterranean Region, by age group, in 2015 (Global Burden of Disease Study 2015, Global, the Eastern Mediterranean Region, 1990–2015)

In 2015, HIV/AIDS caused 10,558 (95% UI 8411–17,775) deaths in the EMR, a tenfold increase from 1990 (936; 470–2226). This equals an increase in age-standardized rate from 0.3 (0.2–0.8) in 1990 to 1.8 (1.4–2.5) per 100,000 population in 2015 (Table 1). HIV/AIDS mortality among males—2.4 (1.8–3.4) deaths per 100,000 population—was double that among females—1.1 (0.9–1.5) deaths per 100,000 population. It affected mostly infants and those aged 25 years or older (Fig. 2). HIV/AIDS deaths as a percentage of all deaths decreased in Kuwait, Lebanon and, Syria at an annualized rate of 3.3, 1.0, and 0.4%, respectively (Table 2). In 2015, the percent of deaths due to HIV/AIDS was highest in Djibouti, and higher than the regional average, 0.2 (0.1–0.2), in Bahrain, Oman, Libya, Lebanon, Saudi Arabia Somalia, Sudan, and UAE. It was lower than the regional average in all remaining countries (Table 2).

**Table 1:** Rates and 95% uncertainty levels (UL) of age-standardized HIV/AIDS mortality per 100,000 population in Eastern Mediterranean Region countries observed in 1990, 2005, and 2015, and expected in 2015 based on Socio-demographic Index (SDI)

Location	1990	2005	2015	
	Rate (95% UL)	Rate (95% UL)	Observed rate (95% UL)	Expected rate based on SDI (SDI)
Eastern Mediterranean Region	0.3 (0.2–0.8)	1.5 (1.2–2.2)	1.8 (1.4–2.5)	35.1 (0.55)
Low and lower middle income				
Afghanistan	0.5 (0.0–2.6)	1.1 (0.1–6.0)	1.0 (0.2–3.5)	45.0 (0.29)
Somalia	2.0 (0.7–4.4)	20.4 (13.4–28.3)	19.1 (13.8–25.0)	44.1 (0.15)
Djibouti	10.6 (2.7–30.0)	82.2 (53.7–115.9)	45.8 (30.8–60.9)	46.2 (0.46)
Egypt	0.0 (0.0–0.0)	0.2 (0.1–0.2)	0.2 (0.2–0.3)	31.2 (0.62)
Morocco	0.1 (0.1–0.1)	0.5 (0.4–0.6)	1.1 (0.8–1.4)	43.6 (0.50)
Pakistan	0.1 (0.0–0.5)	0.3 (0.0–1.7)	0.9 (0.2–3.2)	46.3 (0.47)
Palestine	0.0 (0.0–0.1)	0.3 (0.3–0.4)	0.4 (0.3–0.7)	35.9 (0.57)
Sudan	2.5 (0.7–5.5)	12.4 (8.6–16.6)	13.8 (10.5–16.4)	45.7 (0.43)
Syria	0.2 (0.2–0.2)	0.1 (0.1–0.1)	0.2 (0.1–0.3)	34.8 (0.58)
Tunisia	0.2 (0.0–0.1)	0.3 (0.2–0.3)	0.8 (0.6–1.0)	27.9 (0.65)
Yemen	0.8 (0.0–4.5)	1.5 (0.2–9.3)	0.8 (0.2–3.3)	45.5 (0.41)
Upper middle and high income				
Iran	0.1 (0.0–0.1)	0.4 (0.4–0.5)	0.7 (0.5–1.0)	23.5 (0.72)
Iraq	0.0 (0.0–0.1)	0.2 (0.2–0.2)	0.4 (0.3–0.5)	34.8 (0.58)
Jordan	0.0 (0.0–0.1)	0.2 (0.1–0.2)	0.2 (0.1–0.3)	25.5 (0.70)
Lebanon	2.1 (0.1–17.1)	2.0 (0.2–12.7)	1.7 (0.3–8.2)	17.0 (0.75)
Libya	0.9 (0.0–5.0)	2.1 (0.0–12.9)	1.7 (0.1–9.0)	28.9 (0.64)
Bahrain	0.4 (0.1–0.8)	1.4 (1.0–2.0)	1.3 (0.8–2.2)	15.2 (0.78)
Kuwait	0.4 (0.3–0.4)	0.4 (0.4–0.5)	0.2 (0.2–0.2)	7.2 (0.86)
Oman	0.1 (0.1–0.1)	1.0 (0.9–1.3)	1.5 (1.1–2.0)	21.9 (0.73)
Qatar	0.3 (0.1–0.5)	0.5 (0.3–0.9)	0.3 (0.2–0.6)	12.4 (0.80)
Saudi Arabia	0.6 (0.1–3.1)	1.6 (0.6–5.6)	1.5 (0.7–4.0)	16.9 (0.76)
United Arab Emirates	1.0 (0.0–6.3)	2.6 (0.1–16.7)	2.2 (0.1–11.7)	7.2 (0.88)

Global Burden of Disease 2015 study, Eastern Mediterranean countries, 1990–2015

**Table 2:** Percent of deaths, YLDs, and YLLs attributable to HIV/AIDS, and their relative annual percent change, 1990–2015, Eastern Mediterranean Region countries

Location	% Death	Annual % change	% YLLs	Annual % change	% YLDs	Annual % change	% DALYs	Annual % change
Eastern Mediterranean Region	0.2 (0.1–0.2)	6.7	0.3 (0.2–0.4)	6.7	0.0 (0.0–0.1)	4.7	0.2 (0.2–0.3)	6.6
Low and lower middle income								
Afghanistan	0.0 (0.0–0.2)	2.7	0.1 (0.0–0.2)	2.5	0.0 (0.0–0.1)	1.4	0.1 (0.0–0.2)	2.4
Somalia	1.1 (0.5–1.9)	9.0	1.5 (0.7–2.6)	9.0	0.3 (0.2–0.6)	6.8	1.3 (0.7–2.0)	8.9
Djibouti	3.7 (2.1–5.5)	5.9	5.9 (3.6–8.8)	5.9	0.9 (0.6–1.4)	3.3	4.6 (3.0–6.6)	5.7
Egypt	0.0 (0.0–0.0)	7.7	0.1 (0.0–0.1)	7.7	0.0 (0.0–0.0)	7.4	0.0 (0.0–0.0)	7.7
Morocco	0.1 (0.1–0.2)	11.4	0.3 (0.2–0.4)	11.2	0.0 (0.0–0.1)	7.7	0.2 (0.1–0.2)	10.8
Pakistan	0.1 (0.0–0.3)	10.3	0.1 (0.0–0.4)	10.2	0.0 (0.0–0.1)	8.1	0.1 (0.0–0.3)	10.1
Palestine	0.1 (0.0–0.1)	9.3	0.1 (0.1–0.2)	9.2	0.0 (0.0–0.0)	8.8	0.1 (0.1–0.1)	9.2
Sudan	1.3 (0.9–1.7)	6.8	2.0 (1.4–2.7)	6.7	0.2 (0.1–0.4)	4.3	1.5 (1.1–1.9)	6.6
Syria	0.0 (0.0–0.0)	−0.4	0.0 (0.0–0.1)	−0.3	0.0 (0.0–0.0)	0.4	0.0 (0.0–0.0)	−0.2
Tunisia	0.1 (0.1–0.2)	6.3	0.3 (0.2–0.4)	8.8	0.0 (0.0–0.0)	6.9	0.2 (0.1–0.2)	8.6
Yemen	0.1 (0.0–0.3)	0.1	0.1 (0.0–0.4)	0.0	0.0 (0.0–0.1)	−0.9	0.1 (0.0–0.3)	−0.1
Upper middle and high income								
Iran	0.1 (0.1–0.1)	10.2	0.2 (0.1–0.2)	10.1	0.0 (0.0–0.0)	4.9	0.1 (0.1–0.2)	9.3
Iraq	0.0 (0.0–0.0)	10.1	0.1 (0.0–0.1)	10.1	0.0 (0.0–0.0)	9.8	0.0 (0.0–0.1)	10.1
Jordan	0.0 (0.0–0.1)	6.5	0.1 (0.0–0.1)	6.4	0.0 (0.0–0.0)	5.6	0.0 (0.0–0.1)	6.3
Lebanon	0.3 (0.0–1.4)	−1.0	0.6 (0.1–2.7)	−1.4	0.0 (0.0–0.1)	−2.2	0.3 (0.1–1.4)	−1.5
Libya	0.2 (0.0–1.1)	2.7	0.4 (0.0–2.2)	2.6	0.0 (0.0–0.2)	1.2	0.3 (0.0–1.4)	2.5
Bahrain	0.2 (0.1–0.4)	4.3	0.5 (0.3–0.9)	4.1	0.0 (0.0–0.1)	3.7	0.3 (0.2–0.4)	4.1
Kuwait	0.0 (0.0–0.0)	−3.3	0.1 (0.1–0.1)	−3.4	0.0 (0.0–0.0)	0.4	0.0 (0.0–0.1)	−3.3
Oman	0.2 (0.2–0.3)	10.1	0.5 (0.3–0.6)	9.7	0.0 (0.0–0.1)	8.8	0.3 (0.2–0.4)	9.7
Qatar	0.1 (0.0–0.1)	0.3	0.1 (0.1–0.2)	−0.1	0.0 (0.0–0.0)	−1.3	0.1 (0.0–0.1)	−0.2
Saudi Arabia	0.3 (0.1–0.7)	3.6	0.6 (0.3–1.6)	3.4	0.0 (0.0–0.1)	2.1	0.3 (0.2–0.9)	3.3
United Arab Emirates	0.3 (0.0–1.5)	3.3	0.6 (0.0–3.1)	3.2	0.0 (0.0–0.2)	1.2	0.4 (0.0–1.9)	3.0

Global Burden of Disease 2015 study, Eastern Mediterranean countries, 1990–2015

*YLDs* years lived with disability, *YLLs* years of life lost

### Years of life lost

Years of life lost to HIV/AIDS increased from 49,094 (24,960–117,290) in 1990 to 526,030 (416,745–734,351) in 2015. The rate of YLLs increased as well for the same period from 15.3 (7.6–36.2) to 81.8 (65.3–114.4) per 100,000 population (e-Table 1). E-Table 1 shows these rates for individual countries. The percent of YLLs due to HIV/AIDS decreased in Kuwait, Lebanon, Syria, and Qatar at annualized rates of 3.4, 1.4, 0.3, and 0.1%, respectively (Table 2).

### Years lived with disability

HIV/AIDS accounted for 26,000 (16,440–38,839) YLDs in 2015, a sixfold increase from 3829 (1875–8539) in 1990. The rate increased from 1.3 (0.6–3.1) per 100,000 population in 1990 to 4.4 (2.7–6.6) in 2015 (e-Table 2). E-Table 2 shows these rates for individual countries. The percent of YLDs due to HIV/AIDS decreased in Lebanon, Qatar, and Yemen by annualized rates of 2.2, 1.3, and 0.9%, respectively (Table 2).

HIV/AIDS caused more YLLs than YLDs at all times (e-Fig. 1).

### Disability-adjusted life years

DALYs due to HIV/AIDS increased tenfold between 1990—52,923 (26,913–124,169)—and 2015—552,030 (439,956–768,775). The rate increased from 16.6 (8.4–38.8) to 86.2 (69.2–120.6) per 100,000 population (e-Table 3). E-Table 3 shows these rates for individual countries. The percent of DALYs due to HIV/AIDS decreased in Kuwait, Lebanon, Qatar, Syria, and Yemen by annualized rates of 3.3, 1.5, 0.2, 0.2, and 0.1%, respectively (Table 2).

### Incidence and prevalence

Incidence and prevalence of HIV/AIDS have increased in the EMR since 1990 from 2.9 (2.0–4.9) and 9.1 (5.1–16.4), to 5.3 (3.9–7.9) and 28.4 (22.3–39.8) per 100,000 population, respectively. The highest and lowest incidence for 2015 was observed in Djibouti and Syria, respectively: 90.9 (55.0–142.4) and 0.4 (0.2–0.5). The highest and lowest prevalence for 2015 were observed in Djibouti and Kuwait, respectively: 919.7 (714.8–1161.9) and 0.0 (0.0–0.0) per 100,000 populations. Table 3 presents estimates of incidence and prevalence of HIV/AIDS in EMR countries in 1990, 2005, and 2015.

**Table 3:** Rates and 95% uncertainty levels (UL) of age-standardized incidence and prevalence of HIV/AIDS per 100,000 population

Location	Incidence (95% UL)			Prevalence (95% UL)		
	1990	2005	2015	1990	2005	2015
Eastern Mediterranean Region	2.9 (2.0–4.9)	4.7 (4.0–5.7)	5.3 (3.9–7.9)	9.1 (5.1–16.4)	23.4 (20.0–28.1)	28.4 (22.3–39.8)
Low and lower middle income						
Afghanistan	2.7 (0.2–20.5)	1.2 (0.4–4.2)	2.6 (0.6–7.3)	10.4 (0.7–57.8)	9.3 (1.9–49.0)	14.9 (4.8–46.7)
Somalia	24.7 (13.1–45.8)	47.4 (34.8–62.4)	29.2 (18.7–45.0)	79.5 (41.1–143.5)	359.2 (276.3–466.0)	293.1 (224.8–380.7)
Djibouti	134.5 (58.4–281.3)	115.8 (82.6–159.5)	90.9 (55.0–142.4)	577.6 (198.5–1501.1)	1176.9 (932.3–1472.4)	919.7 (714.8–1161.9)
Egypt	0.3 (0.2–0.5)	0.5 (0.4–0.8)	1.2 (0.7–1.9)	0.0 (0.0–0.0)	0.5 (0.1–1.1)	3.1 (1.6–4.7)
Morocco	1.6 (1.3–1.9)	3.9 (3.2–5.2)	4.7 (3.9–5.9)	0.0 (0.0–0.0)	12.3 (7.6–17.7)	18.6 (10.2–29.5)
Pakistan	0.9 (0.4–4.3)	1.8 (0.9–4.5)	5.6 (1.9–15.0)	0.7 (0.0–4.6)	2.6 (0.4–9.4)	22.3 (6.0–59.6)
Palestine	0.4 (0.2–0.6)	1.3 (0.9–1.9)	2.0 (1.0–3.4)	0.0 (0.0–0.0)	3.2 (1.6–5.1)	7.0 (3.7–12.0)
Sudan	15.8 (6.0–31.2)	29.7 (22.0–38.5)	15.7 (7.2–27.5)	79.2 (25.8–155.2)	197.1 (155.7–249.7)	167.5 (131.9–213.6)
Syria	0.2 (0.2–0.3)	0.2 (0.1–0.2)	0.4 (0.2–0.5)	0.0 (0.0–0.0)	0.0 (0.0–0.0)	0.6 (0.1–1.7)
Tunisia	0.9 (0.6–1.3)	1.7 (1.1–2.6)	3.0 (1.8–5.0)	0.3 (0.1–0.7)	6.5 (3.3–10.5)	16.0 (7.7–26.9)
Yemen	6.4 (3.4–31.1)	4.4 (3.3–8.0)	3.7 (2.1–7.5)	17.3 (2.1–97.8)	11.9 (4.0–41.6)	16.8 (5.0–41.4)
Upper middle and high income						
Iran	2.4 (2.0–3.3)	5.4 (4.6–6.3)	6.9 (5.8–8.2)	0.9 (0.4–1.5)	9.4 (6.6–14.4)	11.4 (7.8–17.8)
Iraq	0.2 (0.1–0.4)	0.7 (0.5–1.2)	1.6 (0.4–3.2)	0.0 (0.0–0.0)	2.0 (1.0–3.2)	7.6 (3.8–13.4)
Jordan	0.2 (0.1–0.4)	0.3 (0.2–0.5)	0.4 (0.1–0.8)	0.0 (0.0–0.0)	0.2 (0.0–0.5)	0.4 (0.0–0.9)
Lebanon	8.8 (1.6–62.0)	2.4 (1.0–7.0)	2.7 (1.0–6.8)	63.0 (4.3–447.4)	34.6 (6.3–175.9)	28.6 (7.0–123.0)
Libya	6.8 (2.2–34.0)	5.4 (2.5–16.5)	5.7 (2.4–16.1)	26.0 (0.3–182.8)	24.4 (1.5–134.4)	29.8 (2.8–84.1)
Bahrain	2.8 (1.5–6.2)	3.0 (2.2–4.3)	4.0 (1.5–7.6)	9.2 (0.3–20.1)	21.0 (9.1–46.2)	28.5 (12.5–54.1)
Kuwait	0.9 (0.5–1.6)	0.4 (0.3–0.6)	0.4 (0.1–0.6)	0.4 (0.0–1.0)	0.0 (0.0–0.0)	0.0 (0.0–0.0)
Oman	1.0 (0.7–1.5)	3.5 (2.6–4.9)	3.1 (2.2–4.1)	1.4 (0.6–2.8)	29.3 (19.3–43.1)	33.7 (18.3–50.5)
Qatar	1.1 (0.4–3.0)	0.3 (0.2–0.6)	0.4 (0.1–0.8)	5.5 (1.3–9.7)	2.5 (0.8–4.7)	0.9 (0.2–1.8)
Saudi Arabia	4.0 (1.4–17.8)	3.3 (2.1–6.3)	3.8 (2.2–6.6)	15.2 (2.5–80.5)	21.1 (11.0–48.4)	28.2 (16.4–51.7)
United Arab Emirates	6.1 (0.8–32.3)	4.0 (0.8–18.4)	4.7 (0.8–15.7)	11.2 (0.2–51.8)	26.0 (1.6–97.2)	36.0 (3.6–155.5)

Global Burden of Disease 2015 study, Eastern Mediterranean countries, 1990–2015

### Risk factors

Unsafe sex and drug use accounted for 74.1 and 18.8% of HIV deaths, 75.3 and 17.5% of HIV YLLs, 71.9 and 21.3% of HIV YLDs, and 75.1 and 17.7% of HIV DALYs, respectively. In Djibouti, where HIV/AIDS mortality was highest in comparison to all other EMR countries, unsafe sex and drug use contributed to 94.4 and 0.4% of deaths related to HIV/AIDS, respectively. On the other hand, in Syria, where HIV/AIDS mortality was lowest, unsafe sex and drug use contributed to 84.5 and 6.4% of deaths related to HIV/AIDS, respectively. Table 4 presents estimates of risk factors contribution to HIV/AIDS deaths, YLLs, YLDs, and DALYs.

**Table 4:** Percent and 95% uncertainty levels (95% UL) of HIV/AIDS deaths, YLLs, YLDs, and DALYs attributed to unsafe sex and drug use

Location	Percent of HIV/AIDS deaths attributable to		Percent of HIV/AIDS YLLs attributable to		Percent of HIV/AIDS YLDs attributable to		Percent of HIV/AIDS DALYs attributable to	
	Unsafe sex (95% UL)	Drug use (95% UL)	Unsafe sex (95% UL)	Drug use (95% UL)	Unsafe sex (95% UL)	Drug use (95% UL)	Unsafe sex (95% UL)	Drug use (95% UL)
Eastern Mediterranean Region	74.1 (69.1–79.0)	18.8 (13.6–24.4)	75.3 (70.7–79.8)	17.5 (12.6–22.9)	71.9 (66.9–76.7)	21.3 (16.3–27.0)	75.1 (70.5–79.7)	17.7 (12.8–23.0)
Low and lower middle income								
Afghanistan	74.4 (66.5–81.1)	16.3 (9.5–24.5)	74.6 (67.0–80.9)	15.7 (9.1–23.6)	74.4 (66.7–81.2)	16.1 (9.3–24.3)	74.6 (67.0–80.9)	15.7 (9.1–23.6)
Somalia	97.3 (95.8–98.3)	0.9 (0.5–1.6)	97.4 (95.8–98.3)	0.9 (0.5–1.5)	97.3 (95.8–98.3)	0.9 (0.5–1.6)	97.4 (95.8–98.3)	0.9 (0.5–1.6)
Djibouti	94.4 (93.1–95.5)	0.4 (0.3–0.4)	94.2 (92.8–95.4)	0.3 (0.3–0.4)	94.4 (93.3–95.4)	0.4 (0.3–0.5)	94.2 (92.8–95.4)	0.3 (0.3–0.4)
Egypt	74.2 (66.5–80.7)	15.8 (9.0–23.9)	74.4 (66.8–80.5)	15.0 (8.7–23.0)	74.2 (66.4–80.6)	15.7 (9.0–23.9)	74.4 (66.9–80.5)	15.1 (8.7–23.0)
Morocco	74.0 (66.1–80.7)	16.1 (9.2–24.2)	74.2 (66.6–80.5)	15.6 (9.0–23.6)	74.0 (66.3–80.5)	15.9 (9.1–24.0)	74.2 (66.6–80.5)	15.6 (9.0–23.6)
Pakistan	65.9 (62.5–69.1)	32.6 (29.3–36.1)	67.5 (64.3–70.7)	30.9 (27.7–34.2)	66.2 (62.9–69.4)	32.3 (28.9–35.5)	67.3 (64.2–70.5)	31.0 (27.8–34.2)
Palestine	74.4 (66.6–81.1)	16.1 (9.3–24.3)	74.5 (67.0–80.9)	15.4 (9.0–23.5)	74.3 (66.6–80.8)	15.9 (9.1–24.0)	74.5 (66.9–80.9)	15.5 (9.0–23.5)
Sudan	74.5 (66.7–81.2)	16.1 (9.3–24.2)	74.6 (66.9–81.1)	15.5 (9.0–23.6)	74.4 (66.6–81.1)	16.1 (9.4–24.4)	74.6 (66.9–81.1)	15.6 (9.0–23.7)
Syria	84.5 (82.1–86.5)	6.4 (5.5–7.7)	84.5 (82.0–86.6)	6.1 (5.2–7.2)	84.5 (82.2–86.6)	6.3 (5.4–7.5)	84.5 (82.0–86.6)	6.1 (5.2–7.2)
Tunisia	77.3 (74.1–80.0)	15.8 (13.4–18.4)	77.8 (74.7–80.5)	15.0 (12.8–17.4)	78.2 (75.2–80.9)	14.8 (12.5–17.3)	77.8 (74.8–80.5)	15.0 (12.7–17.4)
Yemen	91.0 (90.0–92.0)	2.9 (2.5–3.4)	90.8 (89.7–91.8)	2.8 (2.4–3.2)	90.6 (89.5–91.6)	2.9 (2.5–3.3)	90.8 (89.7–91.8)	2.8 (2.4–3.2)
Upper middle and high income								
Iran	14.1 (12.2–16.2)	78.3 (75.8–80.8)	14.3 (12.4–16.5)	77.6 (75.0–80.3)	14.4 (12.4–16.7)	77.3 (74.4–80.0)	14.3 (12.4–16.5)	77.6 (75.0–80.2)
Iraq	74.4 (66.7–80.9)	15.9 (9.2–23.9)	74.5 (66.9–80.7)	15.2 (8.8–23.3)	74.3 (66.8–80.8)	15.8 (9.1–23.8)	74.5 (66.8–80.7)	15.3 (8.9–23.3)
Jordan	81.8 (79.8–83.6)	9.7 (8.5–11.2)	81.9 (79.8–83.7)	9.2 (8.0–10.5)	81.6 (79.6–83.5)	9.6 (8.3–11.0)	81.8 (79.8–83.7)	9.2 (8.0–10.6)
Lebanon	74.2 (66.0–81.0)	16.5 (9.7–24.7)	74.3 (66.6–80.9)	15.9 (9.3–23.8)	74.3 (66.3–81.0)	16.3 (9.4–24.3)	74.3 (66.6–80.9)	15.9 (9.3–23.8)
Libya	74.2 (66.6–80.7)	15.9 (9.2–24.0)	74.4 (66.9–80.5)	15.2 (8.9–23.2)	74.1 (66.4–80.6)	15.7 (9.0–23.9)	74.3 (66.9–80.5)	15.3 (8.9–23.2)
Bahrain	36.6 (33.2–40.3)	57.0 (53.0–60.5)	37.4 (33.9–41.0)	55.8 (52.0–59.4)	37.0 (33.6–40.8)	56.3 (52.5–59.9)	37.3 (33.9–41.0)	55.9 (52.0–59.4)
Kuwait	74.3 (66.8–80.3)	15.3 (8.9–23.1)	74.7 (66.4–80.4)	13.4 (7.4–22.2)	74.2 (66.5–80.6)	15.9 (9.1–24.0)	74.7 (66.5–80.4)	13.5 (7.5–22.2)
Oman	82.8 (80.2–85.3)	7.6 (6.2–8.9)	82.7 (80.1–85.2)	7.3 (6.0–8.5)	82.4 (79.8–84.9)	7.5 (6.2–8.8)	82.6 (80.1–85.2)	7.3 (6.1–8.6)
Qatar	74.6 (66.6–81.5)	16.6 (9.6–25.0)	74.8 (67.1–81.5)	16.0 (9.2–24.0)	74.6 (66.9–81.5)	16.3 (9.4–24.5)	74.7 (67.1–81.5)	16.0 (9.2–24.1)
Saudi Arabia	75.0 (71.4–78.1)	15.5 (13.1–18.5)	75.2 (71.6–78.5)	14.8 (12.5–17.7)	75.0 (71.5–78.1)	15.3 (12.9–18.2)	75.2 (71.6–78.5)	14.9 (12.5–17.7)
United Arab Emirates	74.7 (66.6–81.6)	16.4 (9.7–24.9)	74.9 (67.1–81.2)	15.8 (9.1–24.1)	74.7 (67.0–81.5)	16.1 (9.4–24.3)	74.9 (67.1–81.2)	15.8 (9.1–24.1)

Global Burden of Disease 2015 study, Eastern Mediterranean countries, 1990–2015

*YLDs* years lived with disability, *YLLs* years of life lost, *DALYs* disability-adjusted life years

### Observed versus expected burden

Despite the increase of HIV/AIDS mortality in EMR countries over time, all, but Djibouti had lower observed deaths than expected based on SDI (Table 1). Expected deaths were within the range of uncertainty for the observed deaths in Djibouti (Table 1). Only Djibouti had higher observed YLLs and YLDs than what would have been expected for 2015 based on SDI (e-Tables 1, 2). Expected YLDs were within the range of uncertainty for the observed YLDs in Bahrain, Lebanon, Libya, Saudi Arabia, and the United Arab Emirates (e-Table 2). Expected DALYs were within the range of uncertainty for the observed DALYs in Djibouti and the United Arab Emirates (e-Table 3).

## Discussion

This is the first GBD study to comprehensively examine the burden and trends of HIV/AIDS-related mortality in EMR countries from 1990 to 2015. Our estimates show a tenfold increase in HIV/AIDS mortality rates and other measures of disease burden for the EMR region with most of the HIV/AIDS burden is contributed by the three poorest countries Djibouti, Somalia, and Sudan. These results highlight the expanding nature of the epidemic in the EMR, in contrast to the other global regions (UNAIDS 2016a). They also affirm the epidemiological evidence indicating emerging HIV epidemics within the last two decades such as among MSM in nearly half of EMR countries (Mumtaz et al. 2011, 2014a) and among PWID in over a third of EMR countries (Mumtaz et al. 2014a, b), two populations that are still being criminalized in this region, making epidemic control harder to reach (Simmons 2014; Aaraj and Chrouch 2016). Despite these rapid increases, HIV disease burden in EMR remains at least tenfold lower than HIV/AIDS mortality at the global level, and at all times (Wang et al. 2016b).

These results indicate that EMR countries are not likely to fulfill the Joint United Nations Program on HIV/AIDS (UNAIDS) “90-90-90” target of diagnosing 90% of all people living with HIV/AIDS, providing ART for 90% of those diagnosed, and achieving viral suppression for 90% of those treated, all by 2020 (UNAIDS 2014b). EMR countries are also not likely to reach the fast-track target of ending AIDS by 2030 (UNAIDS 2016a). The striking gap between the expanding disease burden and global targets for reducing this burden highlights the need for EMR countries to strengthen HIV/AIDS voluntary counseling and testing among the most at-risk populations, improve HIV epidemiological surveillance, and scale up ART and comprehensive prevention services.

A major challenge in the EMR is the weak vital registration and epidemiological surveillance systems. People living with HIV are being diagnosed at a late stage of disease progression, thus their chances of accessing treatment and surviving are decreasing. Most HIV infections appear to be detected through routine screening, such as in the context of blood donation, premarital medical tests, and employment, or visa and residency applications (Hermez et al. 2010). Moreover, data on relevant HIV/AIDS indicators, such as the Global AIDS Response Progress Reporting indicators, are limited in many EMR countries, although quality integrated bio-behavioral surveillance surveys (IBBSS) of hard-to-reach populations have already proven possible in over half of EMRO countries (Abu-Raddad et al. 2010; Mumtaz et al. 2011, 2014b). Sustainability of IBBSS rounds in countries where they have been conducted, and implementing them in countries where they have not been conducted, should be a priority.

These results also affirm the evidence indicating low ART coverage in EMR and persistent challenges with the treatment cascade (World Health Organization 2017; UNAIDS 2016b). EMR has the lowest ART coverage globally at a median of 17% in 2015 (UNAIDS 2016b), and did not reach the 2015 midterm regional objective of 50% coverage under the World Health Organization’s (WHO) initiative to end EMR’s HIV treatment crisis (World Health Organization Regional Office for the Eastern Mediterranean 2014).

The effectiveness of highly active ART was manifested in 1995 and became the new standard for HIV care in 1997, making HIV a manageable disease (Carpenter et al. 1997; Palmisano and Vella 2011). Despite this progress, only three EMR countries showed a decrease in HIV/AIDS mortality. These countries can share lessons with the remaining EMR countries to help them control their epidemics. Moreover, our study showed that for most EMR countries, the increase in YLLs exceeded by far the increase in YLDs during the study period. For instance, while YLDs contributed to 4.7% of HIV/AIDS DALYs in EMR countries, they contributed to 8.4% in European countries (Institute for Health Metrics and Evaluation (IHME) 2017). This indicates that HIV survival is very low in EMR countries, affirming the weak and challenged HIV/AIDS response in this region (Abu-Raddad et al. 2013). Even if HIV/AIDS treatment is available, often it is interrupted and patients struggle to survive. Most health care providers are also not well trained to manage HIV/AIDS patients and/or understand their situations (Khosravanifard et al. 2012; Wilder 2008; Anonymous 2012; Thayer 2012; Upham and Mikkelsen 2012; Hedayati-Moghaddam et al. 2012).

Interestingly, the observed burden of HIV/AIDS was lower than expected in most EMR countries based on their SDI. On the surface, this might be sound like good news. However, the burden of HIV/AIDS has been increasing continuously in the EMR despite the decrease in the rest of the world. While SDI is known to be a strong indicator of health outcomes (Wang et al. 2016a), it is possible that the association with HIV/AIDS is modified by other cultural and social factors in the EMR. SDI only deals with socioeconomic inequalities between countries and does not account for other cultural and social norms. For instance, more of the risky behaviors for HIV, such as access to drugs and alcohol, travel, or multiplicity of concurrent relationships might be more common among higher-SDI groups in the EMR. Further, some of the EMR countries have experienced warfare and conflicts, highlighting the difference in the social determinants of HIV in conflict versus non-conflict settings, with HIV morbidity and mortality closely associated with conflicts (Betsi et al. 2006; Mowafi 2011; Wirtz et al. 2014; Robertson and Hoffman 2014; Doocy et al. 2015; Tunçalp et al. 2015; Calam 2016). Some of these include sexual violence and human rights abuses in conflict settings, interruption of treatment due to mass displacement, disruption of health systems, and resource diversions from health to support wars. However, SDI allows comparisons between countries based on similar indicators, and hence is important to use, despite its limitations.

Meanwhile, the poor management and treatment of HIV/AIDS patients is also a persistent issue. EMR policymakers need to devote adequate funds to expand HIV prevention and treatment services even if the leading causes of deaths, YLLs, and YLDs in the EMR are non-communicable, such as ischemic heart disease, diabetes, and road injuries (Mokdad et al. 2014, 2016). These services need to be expanded, particularly among the most at-risk populations. Countries need to put in place active surveillance systems to detect early infections and monitor the epidemic, in addition to delivering health care to those affected. With drug use playing a significant role in HIV transmission in this region, introducing syringe exchange programs should be considered given its proven effectiveness in preventing HIV transmission (Wilson et al. 2015).

Our study might be subjected to several limitations around the estimation of HIV/AIDS burden. These limitations have been previously described (Wang et al. 2016b). In short, our study estimates mortality with HIV/AIDS as the underlying cause of death without accounting for deaths from other non-communicable causes among people living with HIV. Additionally, data are less available for the most recent years, and our models might have missed recent progress, or lack of it, in some countries. Our estimates have not accounted directly for relevant covariates including prevalence of sexually transmitted infections or rates of ART adherence, ART treatment failure, and HIV testing (Wang et al. 2016b).

Our study showed that HIV/AIDS disease burden is increasing in the vast majority of EMR countries, in contrast to the global declining trend. Increased and coordinated efforts are needed in the region to apply lessons from countries that have succeeded in controlling their epidemic to reduce this burden, reverse its trend, and reach global stipulated targets for HIV/AIDS. More affluent EMR countries must consider ways to bring the region’s more disadvantaged countries to the same level of health. These findings highlight the need for EMR countries to strengthen HIV/AIDS voluntary counseling and testing among the most at-risk populations, improve HIV epidemiological surveillance, and scale up ART and comprehensive prevention services.

## Electronic supplementary material

Below is the link to the electronic supplementary material.

Supplementary material 1 (DOCX 25 kb)

Supplementary material 2 (XLSX 28 kb)
