# Supplementary material for: Trends in HIV/AIDS morbidity and mortality in Eastern Mediterranean countries, 1990–2015: findings from the Global Burden of Disease 2015 study
Source: Int J Public Health. 2017 Aug 3;63(Suppl 1):123–36. doi: 10.1007/s00038-017-1023-0 (PMC5702264; doi:10.1007/s00038-017-1023-0)
Supplement: Supplementary file 1 — Supplementary material 1 (DOCX 25 kb) [file 38_2017_1023_MOESM1_ESM.docx]

Electronic Supplementary Material

**Article title:**

Trends in HIV/AIDS morbidity and mortality in Eastern Mediterranean countries, 1990–2015: Findings from the Global Burden of Disease 2015 study

**Journal:**

International Journal of Public Health

**Authors:**

GBD 2015 Eastern Mediterranean Region HIV/AIDS Collaborators

**Corresponding author:**

Ali H. Mokdad

Institute for Health Metrics and Evaluation, University of Washington, Seattle, WA, United States

Email: mokdaa@uw.edu

e-Table 1: Rates and 95% uncertainty levels (UL) of age-standardized years of life lost due to HIV/AIDS mortality per 100,000 population in Eastern Mediterranean Region countries observed in 1990, 2005, and 2015, and expected in 2015 based on Socio-demographic Index(SDI). (Global Burden of Disease 2015 study, Eastern Mediterranean Countries, 1990-2015).

|  | **1990** | **2005** | **2015** | |
| --- | --- | --- | --- | --- |
| **Location** | **Rate (95% UL)** | **Rate (95% UL)** | **Observed rate (95% UL)** | **Expected rate based on SDI** |
| Eastern Mediterranean Region | 15.3 (7.6 – 36.2) | 72.3 (55.7 – 101.3) | 81.9 (65.3 – 114.4) | 1664.9 |
| **Low and lower middle income** | | | | |
| Afghanistan | 22.0 (0.5 – 117.5) | 46.3 (5.3 – 255.6) | 41.2 (8.5 – 150.2) | 2165.3 |
| Somalia | 96.1 (34.5 – 206.1) | 974.0 (628.2 – 1358.6) | 915.9 (670.7 – 1218.2) | 2162.5 |
| Djibouti | 509.0 (135.6 – 1394.7) | 3894.9 (2566.1 – 5527.3) | 2218.8 (1474.5 – 2999.1) | 2176.4 |
| Egypt | 1.7 (0.8 – 2.1) | 7.9 (6.2 – 9.4) | 11.3 (9.2 – 14.1) | 1478.7 |
| Morocco | 2.9 (2.5 – 3.3) | 23.0 (19.8 – 27.2) | 46.5 (35.7 – 60.6) | 2052.3 |
| Pakistan | 3.2 (0.1 – 23.8) | 13.1 (1.6 – 71.3) | 41.3 (7.0 – 145.6) | 2178.1 |
| Palestine | 1.9 (0.8 – 3.8) | 13.2 (11.3 – 15.5) | 19.5 (14.4 – 28.7) | 1694.1 |
| Sudan | 116.2 (31.0 – 250.8) | 565.7 (388.8 – 752.9) | 621.9 (460.2 – 763.8) | 2161.7 |
| Syria | 8.4 (7.6 – 9.3) | 3.8 (2.6 – 5.6) | 7.8 (3.0 – 15.7) | 1641.7 |
| Tunisia | 3.7 (1.7 – 5.5) | 13.0 (10.8 – 15.7) | 33.3 (24.8 – 43.4) | 1324.8 |
| Yemen | 36.2 (0.7 – 219.9) | 64.7 (6.4 – 390.7) | 36.0 (7.1 – 140.3) | 2157 |
| **Upper middle and high income** | | | | |
| Iran | 2.2 (0.5 – 3.8) | 18.4 (15.8 – 21.1) | 27.2 (21.6 – 37.8) | 1118.9 |
| Iraq | 1.4 (0.5 – 2.8) | 9.4 (7.9 – 10.9) | 17.1 (13.1 – 23.7) | 1644.5 |
| Jordan | 1.6 (0.6 – 3.2) | 8.0 (6.5 – 10.7) | 7.8 (5.0 – 12.7) | 1212.6 |
| Lebanon | 95.1 (4.7 – 744.7) | 89.4 (8.7 – 549.9) | 66.8 (11.5 – 320.3) | 803.2 |
| Libya | 40.6 (0.1 – 227.2) | 94.1 (2.1 – 567.4) | 76.8 (3.4 – 406.1) | 1369 |
| Bahrain | 19.5 (6.7 – 35.3) | 59.2 (44.2 – 86.6) | 54.7 (36.6 – 89.7) | 711.1 |
| Kuwait | 18.8 (16.8 – 20.9) | 19.5 (17.7 – 21.4) | 7.9 (7.1 – 8.8) | 332 |
| Oman | 5.4 (4.7 – 6.4) | 47.5 (39.5 – 61.2) | 61.7 (47.8 – 80.8) | 1029.3 |
| Qatar | 12.2 (4.1 – 21.1) | 22.7 (14.5 – 37.4) | 11.9 (6.6 – 23.1) | 571.3 |
| Saudi Arabia | 27.8 (4.4 – 148.3) | 69.6 (28.3 – 253.8) | 64.7 (33.0 – 171.1) | 792.6 |
| United Arab Emirates | 42.4 (0.1 – 287.4) | 110.6 (2.6 – 716.8) | 93.2 (4.3 – 494.8) | 327.8 |

e-Table 2: Rates and 95% uncertainty levels (UL) of age-standardized years of life lived with disability due to HIV/AIDS mortality per 100,000 population in Eastern Mediterranean Region countries observed in 1990, 2005, and 2015, and expected in 2015 based on Socio-demographic Index(SDI). (Global Burden of Disease 2015 study, Eastern Mediterranean Countries, 1990-2015).

|  | **1990** | **2005** | **2015** | |
| --- | --- | --- | --- | --- |
| **Location** | **Rate (95% UL)** | **Rate (95% UL)** | **Observed rate (95% UL)** | **Expected rate based on SDI** |
| Eastern Mediterranean Region | 1.3 (0.6 – 3.1) | 3.7 (2.3 – 5.6) | 4.4 (2.7 – 6.6) | 36.7 |
| **Low and lower middle income** | | | | |
| Afghanistan | 1.7 (0.1 – 10.9) | 2.2 (0.2 – 11.5) | 2.4 (0.5 – 8.6) | 134.3 |
| Somalia | 8.3 (3.4 – 18.9) | 47.3 (24.4 – 88.1) | 45.1 (24.1 – 81.8) | 217.3 |
| Djibouti | 51.0 (13.9 – 148.6) | 175.2 (91.3 – 325.5) | 115.2 (71.4 – 182.0) | 51 |
| Egypt | 0.1 (0.1 – 0.3) | 0.6 (0.2 – 1.1) | 0.9 (0.4 – 1.6) | 17.1 |
| Morocco | 0.5 (0.3 – 0.7) | 2.0 (1.0 – 3.5) | 3.4 (1.4 – 6.9) | 40.6 |
| Pakistan | 0.4 (0.1 – 2.1) | 0.9 (0.3 – 4.0) | 3.0 (0.7 – 9.1) | 51.2 |
| Palestine | 0.2 (0.1 – 0.4) | 0.9 (0.4 – 2.0) | 1.4 (0.6 – 3.1) | 24.6 |
| Sudan | 8.8 (1.6 – 20.9) | 25.7 (14.5 – 43.3) | 25.7 (14.3 – 43.8) | 65.1 |
| Syria | 0.4 (0.2 – 1.0) | 0.2 (0.1 – 0.5) | 0.5 (0.1 – 1.2) | 22.7 |
| Tunisia | 0.4 (0.2 – 0.5) | 1.1 (0.5 – 1.9) | 2.4 (0.9 – 4.8) | 13.1 |
| Yemen | 3.0 (0.8 – 14.7) | 3.1 (0.9 – 14.9) | 2.4 (0.8 – 7.5) | 73.1 |
| **Upper middle and high income** | | | | |
| Iran | 0.7 (0.5 – 1.1) | 1.9 (1.2 – 2.9) | 2.5 (1.5 – 4.2) | 8.6 |
| Iraq | 0.1 (0.0 – 0.2) | 0.7 (0.3 – 1.4) | 1.2 (0.5 – 2.6) | 22.7 |
| Jordan | 0.1 (0.0 – 0.3) | 0.5 (0.2 – 1.1) | 0.5 (0.2 – 1.1) | 10.1 |
| Lebanon | 6.3 (0.5 – 44.3) | 4.4 (0.6 – 25.9) | 3.7 (0.7 – 15.7) | 6 |
| Libya | 3.4 (0.6 – 14.5) | 5.0 (0.7 – 28.3) | 4.5 (0.7 – 18.4) | 14.1 |
| Bahrain | 1.4 (0.5 – 3.4) | 3.4 (1.1 – 8.7) | 3.5 (1.3 – 7.6) | 5.5 |
| Kuwait | 0.3 (0.1 – 0.8) | 0.5 (0.1 – 1.5) | 0.4 (0.1 – 1.0) | 2.5 |
| Oman | 0.4 (0.2 – 0.9) | 3.3 (1.8 – 5.5) | 3.9 (1.6 – 7.7) | 8.8 |
| Qatar | 1.0 (0.3 – 2.2) | 1.3 (0.3 – 3.5) | 0.7 (0.2 – 1.9) | 4.3 |
| Saudi Arabia | 2.2 (0.5 – 11.5) | 3.8 (1.3 – 11.4) | 3.8 (1.7 – 8.8) | 6.3 |
| United Arab Emirates | 3.8 (0.2 – 23.4) | 5.8 (0.3 – 32.0) | 5.0 (0.3 – 22.7) | 2.9 |

e-Table 3: Rates and 95% uncertainty levels (UL) of age-standardized years of disability-adjusted life years due to HIV/AIDS mortality per 100,000 population in Eastern Mediterranean Region countries observed in 1990, 2005, and 2015, and expected in 2015 based on Socio-demographic Index(SDI). (Global Burden of Disease 2015 study, Eastern Mediterranean Countries, 1990-2015).

|  | **1990** | **2005** | **2015** | |
| --- | --- | --- | --- | --- |
| **Location** | **Rate (95% UL)** | **Rate (95% UL)** | **Observed rate (95% UL)** | **Expected rate based on SDI** |
| Eastern Mediterranean Region | 16.6 (8.4 – 38.8) | 75.9 (58.8 – 106.4) | 86.2 (69.2 – 120.6) | 1701.6 |
| **Low and lower middle income** | | | | |
| Afghanistan | 23.7 (0.6 – 128.8) | 48.5 (5.7 – 264.4) | 43.5 (8.9 – 156.5) | 2299.7 |
| Somalia | 104.4 (39.0 – 222.5) | 1021.3 (676.7 – 1420.8) | 961.0 (708.4 – 1274.5) | 2379.9 |
| Djibouti | 560.0 (157.4 – 1508.8) | 4070.1 (2771.2 – 5695.5) | 2334.0 (1582.6 – 3125.5) | 2227.4 |
| Egypt | 1.8 (0.9 – 2.3) | 8.4 (6.4 – 10.5) | 12.2 (9.6 – 15.7) | 1495.8 |
| Morocco | 3.4 (2.9 – 3.9) | 25.0 (21.7 – 30.0) | 49.8 (37.4 – 67.5) | 2092.9 |
| Pakistan | 3.6 (0.3 – 26.7) | 14.1 (1.9 – 75.8) | 44.3 (7.8 – 155.2) | 2229.3 |
| Palestine | 2.1 (0.9 – 4.0) | 14.1 (11.8 – 17.3) | 21.0 (15.1 – 31.9) | 1718.7 |
| Sudan | 125.0 (36.7 – 267.8) | 591.4 (416.6 – 778.4) | 647.5 (492.3 – 785.3) | 2226.8 |
| Syria | 8.8 (7.7 – 10.2) | 4.0 (2.7 – 6.0) | 8.3 (3.1 – 16.8) | 1664.4 |
| Tunisia | 4.1 (2.0 – 5.8) | 14.1 (11.8 – 16.8) | 35.8 (25.8 – 47.5) | 1337.9 |
| Yemen | 39.1 (1.7 – 232.1) | 67.8 (7.8 – 399.6) | 38.4 (8.0 – 148.1) | 2230 |
| **Upper middle and high income** | | | | |
| Iran | 2.9 (1.2 – 4.6) | 20.3 (17.7 – 23.5) | 29.7 (23.5 – 42.0) | 1127.6 |
| Iraq | 1.5 (0.6 – 3.0) | 10.1 (8.4 – 12.2) | 18.3 (13.9 – 26.1) | 1667.3 |
| Jordan | 1.7 (0.7 – 3.4) | 8.5 (6.7 – 11.7) | 8.3 (5.2 – 14.0) | 1222.7 |
| Lebanon | 101.4 (5.3 – 785.3) | 93.8 (9.6 – 573.5) | 70.5 (12.5 – 334.5) | 809.2 |
| Libya | 44.0 (0.8 – 238.9) | 99.0 (2.8 – 600.7) | 81.3 (4.2 – 422.6) | 1383.1 |
| Bahrain | 20.9 (8.3 – 37.3) | 62.6 (45.5 – 93.5) | 58.2 (38.0 – 97.3) | 716.6 |
| Kuwait | 19.1 (17.1 – 21.2) | 20.0 (18.1 – 22.2) | 8.3 (7.4 – 9.4) | 334.5 |
| Oman | 5.9 (5.1 – 6.9) | 50.8 (43.0 – 63.7) | 65.6 (49.9 – 87.1) | 1038 |
| Qatar | 13.1 (4.7 – 22.6) | 23.9 (14.8 – 40.6) | 12.6 (6.8 – 25.5) | 575.6 |
| Saudi Arabia | 30.0 (5.0 – 159.5) | 73.4 (29.8 – 266.2) | 68.5 (35.1 – 180.8) | 798.9 |
| United Arab Emirates | 46.1 (0.4 – 320.2) | 116.4 (2.9 – 732.7) | 98.3 (4.7 – 522.2) | 330.7 |

e-Figure 1: Distribution of age-standardized years of life lost and years lived with disability due to HIV/AIDS, Eastern Mediterranean Region countries, 1990–2015. (Global Burden of Disease 2015 study, Eastern Mediterranean Region, 1990-2015

).
